# Emerging biomaterials for osteomyelitis treatment: from material design to anti-infection and tissue regeneration

**DOI:** 10.3389/fcell.2026.1922621

**Published:** 2026-08-03

**Authors:** Shi-Jiu Yin, Yu Chen, Jia Li, Hai Yang, Yi Ren, Biao Cao, Qi-Rui Geng, Jun-Qi Niu, Heng Gong, Hui Zhang

**Affiliations:** 1 Department of Orthopedic Surgery and Orthopedic Research Institute, West China Hospital, Sichuan University, Chengdu, Sichuan, China; 2 Department of Orthopaedic Surgery, Shangjin Nanfu Hospital, Chengdu, Sichuan, China

**Keywords:** antibacterial therapy, biomaterials, bone regeneration, drug delivery, immunomodulation, osteomyelitis

## Abstract

Osteomyelitis (OM) is an infectious disease caused by the invasion of bone tissue by pathogenic microorganisms. It is frequently associated with biofilm formation, residual sequestra, local ischemia and hypoxia, elevated oxidative stress, and immune dysfunction. Conventional treatments rely on systemic antibiotics, surgical debridement, antibiotic-loaded bone cement, and bone transport techniques. Although these approaches can control infection in some cases, they remain limited by insufficient drug penetration, nondegradable materials, the need for secondary surgery, difficulty in repairing bone defects, and the risk of recurrence. In recent years, emerging biomaterials have provided new therapeutic strategies for OM by enabling the local delivery of antimicrobial agents, antimicrobial peptides, metal ions, or gaseous molecules, often in combination with photothermal therapy, sonodynamic therapy, pH/enzyme-responsive systems, and immunomodulation. The aim of this mini-review is to briefly summarize the pathological features of OM, outline the major design strategies of biomaterials currently used for OM treatment, and discuss the challenges associated with their clinical translation.

## Highlights

- This review summarizes the complex pathological microenvironment of osteomyelitic lesions, including biofilm formation, sequestra, ischemia and hypoxia, oxidative stress, and immune imbalance.
- This review outlines the applications of hydrogels, nano-delivery systems, 3D-printed scaffolds, and related biomaterials in local antibacterial therapy, antibiofilm treatment, and responsive drug release.
- This review emphasizes that emerging biomaterials should evolve from single anti-infective functions toward integrated therapeutic strategies combining infection control, immunomodulation, angiogenesis, and bone regeneration.

## 1 Introduction

Osteomyelitis (OM) is an infectious disease that occurs following hematogenous infection, open trauma, internal fixation of fractures, or other iatrogenic procedures. *Staphylococcus aureus* and its associated drug-resistant strains are among the most important causative pathogens (Woods et al., 2021; Urish and Cassat, 2020). Recent epidemiological data suggest that the disease burden associated with OM continues to increase. Inpatient data from Germany and a large multicenter database from the United States both indicate that the prevalence or incidence of OM remains clinically significant (Walter et al., 2021; Aljadani et al., 2025). Beyond the infection itself, OM can lead to long-term pain, impaired motor function, and reduced quality of life. In some patients, even after the infection has been controlled, limb function may still fail to return to normal levels (Norris et al., 2021; Rodham et al., 2023).

**FIGURE 1:**
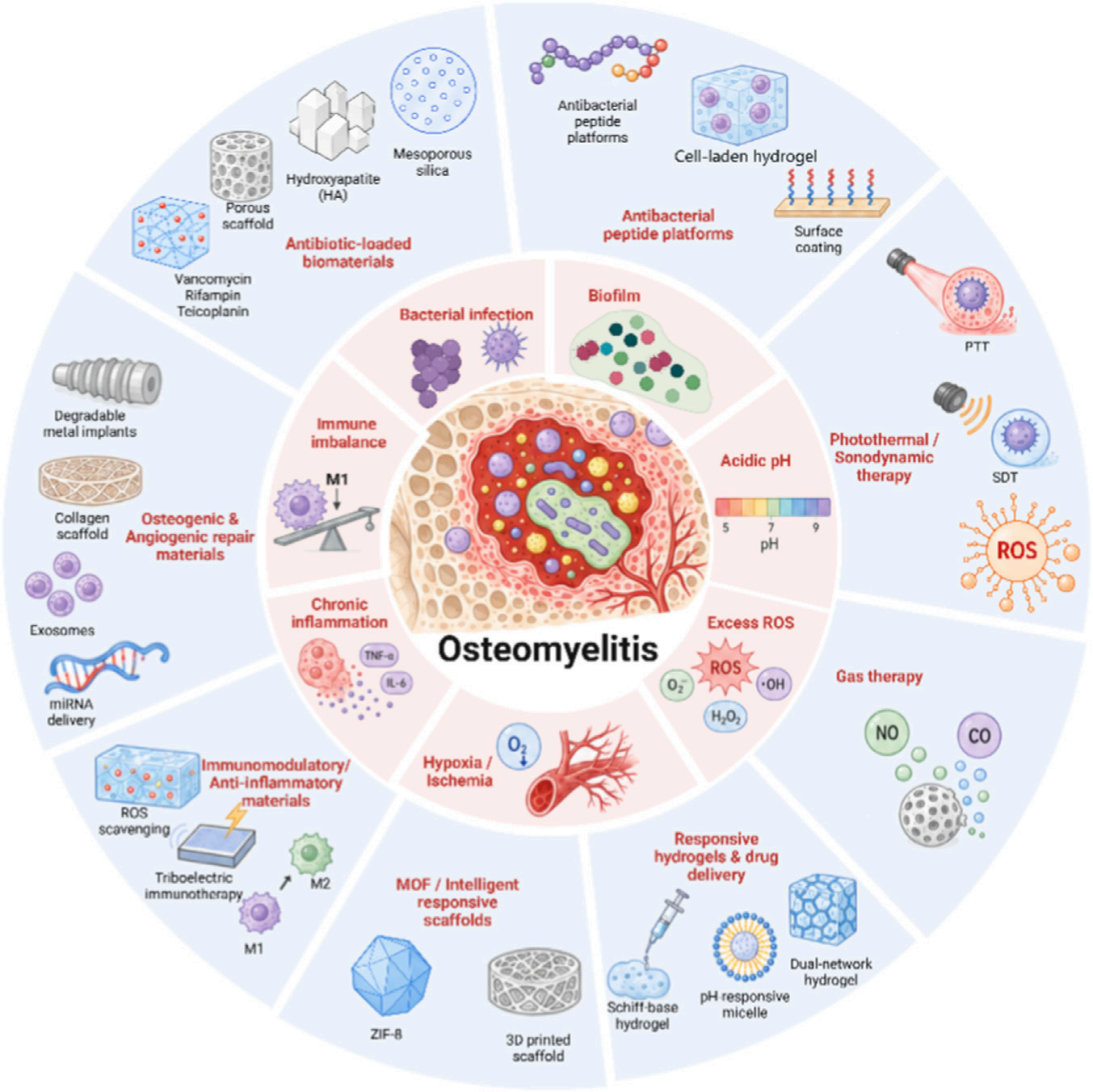
Schematic overview of the osteomyelitis microenvironment and biomaterial-based therapeutic strategies.

The fundamental reason why OM is difficult to treat lies in the fact that the lesion is not merely a bacterial infection, but rather a complex pathological microenvironment composed of infection, necrosis, ischemia, inflammation, and bone defects. Antibacterial therapy alone is insufficient to simultaneously address bacterial eradication, sequestrum removal, microenvironmental restoration, and bone regeneration. Therefore, emerging biomaterials capable of achieving local multifunctional synergy are gradually becoming an important research direction for OM treatment. This mini-review focuses primarily on the core rationale and representative strategies underlying recent biomaterial design, emphasizing their potential value in the therapeutic process of first controlling infection and subsequently promoting bone regeneration in OM (Figure 1).

## 2 Pathological microenvironment of OM and the rationale for biomaterial design

The typical features of chronic OM include biofilm formation, residual sequestra, and impaired local blood supply. After adhering to necrotic tissue or implant surfaces, bacteria can secrete extracellular matrix and form biofilms. Biofilms not only restrict antibiotic penetration but also maintain bacteria in a low-metabolic or tolerant state, thereby increasing the risk of recurrence (Masters et al., 2019). Meanwhile, infection leads to the destruction of trabecular bone and bone matrix, while sequestra and occlusion of surrounding blood vessels further impair the ability of immune cells and therapeutic agents to access the lesion site (Lew and Waldvogel, 2004; Panteli and Giannoudis, 2016). The emergence of multidrug-resistant bacteria also places increasing pressure on conventional antibacterial strategies (Subramanyam et al., 2023).

At the microscopic level, OM lesions are often accompanied by hypoxia, nutrient deprivation, reactive oxygen species (ROS) accumulation, and chronic inflammation. Excessive ROS can damage cellular proteins, DNA, and mitochondrial function, while also inhibiting bone formation (Massaccesi et al., 2022). Persistent inflammatory stimulation may lead to macrophage dysfunction and M1 polarization, further exposing tissues to a sustained inflammatory microenvironment and suppressing tissue repair (Seebach and Kubatzky, 2019; Luo et al., 2024). Therefore, OM-related biomaterials should not only eradicate pathogenic microorganisms within the lesion but also be systematically designed to modulate the local microenvironment. Such materials should enhance local antibacterial efficacy and disrupt biofilms, reduce oxidative stress and chronic inflammation, restore or promote angiogenesis, and induce osteogenesis to achieve complete bone defect repair and regeneration.

## 3 Limitations of conventional treatments and opportunities for biomaterial intervention

Current clinical treatment strategies commonly include systemic antibiotic administration, surgical debridement, local antibiotic carriers, and bone transport techniques. Antibiotic therapy needs to be adjusted according to pathogenic identification and antimicrobial susceptibility testing; however, insufficient blood supply within OM lesions can limit the effective concentration achieved by systemic administration (Mader et al., 1999). Thorough debridement can remove necrotic and infected tissues and is an important prerequisite for controlling chronic infection, but excessive debridement may result in large segmental bone defects and soft-tissue defects (Metsemakers et al., 2018). Antibiotic-loaded bone cement can release high concentrations of antibiotics locally and represents an important conventional approach for local therapy (Gogia et al., 2009). However, commonly used polymethyl methacrylate (PMMA) is nondegradable and often requires a second surgery for removal. In addition, its antibiotic release profile is characterized by an early burst release followed by a subsequent decline in drug concentration. Some studies have even shown that biofilms may still form on the material surface after its antibacterial efficacy diminishes (Ford and Cassat, 2017; van Vugt et al., 2019). Bone transport is suitable for large segmental bone defects, but it requires a prolonged period of external fixation and imposes a substantial care burden on patients (Feng et al., 2023).

These limitations create opportunities for the development of biodegradable, multifunctional, and locally responsive biomaterials. Ideally, such materials should fill bone defects after debridement, provide sustained infection control, and gradually shift from antibacterial activity toward immunomodulation and bone regeneration as the disease course evolves.

## 4 Major strategies of emerging biomaterials

### 4.1 Antibacterial and antibiofilm materials

Representative biomaterial-based strategies for OM treatment, including their antibacterial mechanisms and regenerative or immunomodulatory effects, are summarized in Table 1. Local antibacterial therapy remains the foundation of biomaterial design for OM. Platforms such as hydrogels, porous metals, hydroxyapatite, mesoporous silica, and 3D-printed scaffolds can be loaded with antibacterial agents, including vancomycin, rifampicin, and teicoplanin, to achieve high local drug concentrations and sustained release (Sun et al., 2024; Gupta et al., 2024; Nie et al., 2022; Aguilera-Correa et al., 2022; Sheehy et al., 2025; Qian et al., 2023). Compared with systemic administration, local delivery can reduce systemic drug toxicity and maintain effective drug exposure in lesions with poor blood supply. Antimicrobial peptides, as proteinaceous peptides with potent antibacterial activity, exhibit membrane-disruptive effects, intracellular targeting capability, and a low propensity to induce drug resistance. They have also been incorporated into 3D-printed scaffolds, surface coatings, and cell/hydrogel composite systems (Zhang et al., 2024; Chen et al., 2025). In addition, non-pharmacological antibacterial strategies have attracted increasing attention in recent years. Photothermal materials can generate heat under near-infrared irradiation and damage bacteria, whereas sonodynamic materials can utilize the deep tissue penetration of ultrasound to produce reactive oxygen species for bacterial killing. The combination of these two approaches can enhance the eradication of biofilms and drug-resistant bacteria (Cheng et al., 2022; Song et al., 2025).

**TABLE 1:** Representative biomaterial-based strategies for osteomyelitis treatment.

Therapeutic cargo	Carrier/platform	Target bacteria	Antibacterial action	Regenerative/immune effect	Model	Ref.	Year
Vancomycin	PEG/ODEX *in situ* hydrogel	*S. aureus*	Sustained local release; cell-wall inhibition	Infection/inflammation control	Rat femoral defect	Sun et al. (2024)	2024
Nano-hydroxyapatite (nHA)	CS/QCS thermosensitive hydrogel	*S. aureus*; *E. coli*	Cationic QCS membrane disruption	Osteogenic differentiation	Rabbit bone defect	Tian et al. (2024)	2024
EGCG-MoS_2_ microspheres	HAMA hydrogel (EMgel)	MRSA; *S. aureus*; *E. coli*	US-triggered ROS/EGCG release	Anti-inflammatory osteogenesis; upregulated OCN	Rat tibial OM	Guo et al. (2024)	2024
Rifampicin + exosomes	Hydroxyapatite nanocement	*S. aureus*	Local rifampicin release; RNA-synthesis inhibition	Mineralization and periosteal repair	Rat tibial OM	Gupta et al. (2024)	2024
LL37-producing MSCs	OCAHM Mg hydrogel	*S. aureus*	Continuous LL37 secretion; membrane disruption	Mg-assisted osteogenesis; M2 shift	Mouse tibial OM	Chen et al. (2025)	2025
Ciprofloxacin	Bone-targeting pH/charge-switchable micelles	MRSA; *S. aureus*	pH-enhanced adhesion/penetration; DNA inhibition	Infection/inflammation control	Rat tibial OM	Lu et al. (2025)	2025
Methicillin + NO donor	Targeted NO-donor system	MRSA	NO biofilm disruption; methicillin resensitization	Anti-inflammatory bone repair	Rat tibial OM	Lv et al. (2025)	2025
LL37 + CO-generating CONPs	GelMA microspheres (BRDMs)	MRSA	LL37 capture/lysis; ROS-triggered CO release	Reduced inflammation; BMSC osteogenesis	Rat femoral defect	Zhuang et al. (2024)	2024
Au/TNT@PG	Guanidinium-rich Au/TNT nanotubes	MRSA	Biofilm capture/penetration; US-induced ROS	M2 polarization; osteoblast activation	Rat tibial OM	Cheng et al. (2022)	2022
Au/PDA/HRP@DLP (APH@DLP)	DOTAP/DOPE cationic liposomes	*S. aureus*	PTT/SDT heat-ROS synergy	Adaptive immunity and tissue repair	Chronic osteomyelitis model	Song et al. (2025)	2025
M-Fe_3_O_4_/Au NPs	RAW 264.7 macrophage membrane	*S. aureus*	MW-triggered heat/superoxide; membrane damage	M2 polarization; MSC osteogenesis	Rabbit tibial defect	Fu et al. (2021)	2021
Drug-free electrical stimulation	Pulsed DC stimulation platform	*S. aureus*	Direct electrical killing; M1 antibacterial activation	Immune recruitment/memory; bone healing	Rat cranial defect	Yuan et al. (2024)	2024
TA-D-tyrosine NPs	Konjac/gelatin coating on Ti implant	*S. aureus*	PTT sensitization; biofilm dispersal; TA killing	ROS scavenging; M2/MSC osteogenesis	Rat femoral implant	Ding et al. (2023)	2023
Zn/Li/Ag ions	Biodegradable Zn-Li-Ag alloy implant	MRSA	Metabolic interference; ROS induction	Osteogenic support; bone retention	Rat femoral implant	Jia et al. (2022)	2022
Zn^2+^/Ag^+^ + tannic acid	ZnO-TA-Ag porous scaffold	*S. aureus*; *E. coli*	Fast dual-metal ion release	ROS scavenging; BMSC/M2 osteogenesis	Rat femoral condyle defect	Zhao et al. (2023)	2023
Vancomycin	GelMA/PLGA in porous Ta scaffold	MRSA	Sustained release; cell-wall inhibition	Ta/GelMA-supported osteointegration	Rat femoral defect	Qian et al. (2023)	2023
ZIF-8@CuO	3D-printed PLGA scaffold	*S. aureus*; *E. coli*	pH/NIR Cu^2+^ release; ROS/PTT	Zn^2+^-driven osteogenesis; ROS control	Rat cranial defect	Li et al. (2025b)	2025b
MCFS nanosheets	3D-printed bioactive-glass scaffold	MRSA	Metal-ion metabolic inhibition; NIR-II PTT	TGF/BMP/RUNX2/VEGF osteogenesis	Rabbit tibial defect	Bian et al. (2024)	2024
Cu^2+^	Cu-doped bioactive glass (CuBG)	*S. aureus*	Local Cu^2+^ antibacterial activity	Osteogenesis and angiogenesis	Chick CAM model	Ryan et al. (2019)	2019
AntagomiR-138 + CuBG NPs	Cu-doped bioactive glass	*S. aureus*; *E. coli*	Cu^2+^ oxidative stress; cell-wall damage	miR-138 inhibition; hMSC osteogenesis	Rat femoral defect; CAM	Sadowska et al. (2024)	2024
Vancomycin + gentamicin	Collagen-hydroxyapatite scaffold	*S. aureus*	Collagenase-responsive antibiotic release	Scaffold-supported osteogenesis	Mouse tibial OM	Sheehy et al. (2025)	2025
HHC-36 AMP + simvastatin	HAMA/GelMA hydrogel on PLA-mPEG scaffold	*S. aureus*; *E. coli*	AMP membrane disruption; trap-mediated clearance	BMP-2/Smad osteogenesis/angiogenesis	Rat cranial defect	Zhang et al. (2024)	2024
Vancomycin + polymyxin B	CA-Mg/BSA-GP dual-network hydrogel	MRSA; *E. coli*	CA-assisted antibiotic eradication of MDR bacteria	Bacteria/factor adsorption; Mg osteogenesis	Rat tibial defect	Yu et al. (2026)	2026
Magainin II + CNBIS + BMSCs	COL1MA hydrogel	MRSA	Sustained Magainin II release	Biomineralized niche; vascular/neural bone repair	Rabbit femoral defect	Deng et al. (2025)	2025
Piezoelectric catalyst	T-BSTO-Vo	MRSA	US-activated ROS and piezoelectric charges	PI3K-AKT/Ca^2+^ innervated osteogenesis	Rat tibial defect	Xu et al. (2025)	2025

Terms are abbreviated for readability; full bibliographic information is provided in the References section. AMP, antimicrobial peptide; BMSC, bone marrow mesenchymal stem cell; CAM, chorioallantoic membrane; OM, osteomyelitis; PTT, photothermal therapy; SDT, sonodynamic therapy; US, ultrasound.

Furthermore, gas therapies based on nitric oxide and carbon monoxide can interfere with bacterial respiration or disrupt biofilm structures, providing alternative strategies for drug-resistant infections (Zhuang et al., 2024). However, physical and gas-based therapies may also pose risks such as local thermal injury, excessive ROS generation, and difficulty in dose control. Therefore, a careful balance must be achieved between antibacterial potency and tissue safety.

### 4.2 Microenvironment-responsive and drug delivery systems

Because OM lesions are characterized by specific microenvironmental features, such as low pH, elevated ROS levels, and enrichment of inflammatory factors, responsive delivery systems can improve therapeutic precision. Hydrogels formed through Schiff-base reactions, ionic crosslinking, or thermosensitive gelation can degrade or alter their network structures within the infectious microenvironment, thereby enabling on-demand release (Sun et al., 2024; Li K. et al., 2025; Tian et al., 2024; Yu et al., 2026). Dual-network hydrogels can also simultaneously adsorb bacteria and inflammatory factors, reducing pathogen burden while improving the local inflammatory state (Yu et al., 2026).

Nanodelivery systems have further expanded the range of therapeutic agents available for OM treatment. Bone-targeting nanoparticles, mesoporous silica, liposomes, amphiphilic micelles, and metal–organic framework materials can deliver antibiotics, gas precursors, osteogenic small molecules, or nucleic acid therapeutics (Nie et al., 2022; Aguilera-Correa et al., 2022; Guo et al., 2024; Lv et al., 2025; Lu et al., 2025; Li W. et al., 2025; Sadowska et al., 2024). For example, pH-responsive micelles can release antibiotics in infected regions and eradicate biofilms, whereas ZIF-8-based materials can disassemble under acidic conditions and release metal ions and antibacterial components, enabling a functional transition from early-stage antibacterial activity to late-stage tissue repair (Lu et al., 2025; Li W. et al., 2025). In addition, nucleic acid therapeutics, such as miRNA inhibitors, can be locally protected and sustainably released by scaffolds, thereby relieving osteogenic inhibition and promoting the repair of infected bone defects (Sadowska et al., 2024).

### 4.3 Immunomodulatory and anti-inflammatory materials

The progression of OM is not determined solely by bacterial burden; the host immune status also plays a critical role in disease outcomes. Persistent M1-like inflammation can exacerbate tissue destruction, whereas appropriate induction of an M2-like reparative phenotype is beneficial for angiogenesis and bone regeneration (Seebach and Kubatzky, 2019; Luo et al., 2024). Therefore, immunomodulatory materials commonly reshape the lesion microenvironment by scavenging ROS, adsorbing inflammatory factors, regulating macrophage polarization, or activating adaptive immunity. Representative strategies include anti-inflammatory hydrogel coatings, microwave-excited pseudo-macrophage materials, antimicrobial peptide-producing cell hydrogels, and drug-free triboelectric immunotherapy devices (Chen et al., 2025; Ding et al., 2023; Fu et al., 2021; Yuan et al., 2024). These materials are no longer limited to direct bacterial killing; instead, they reduce the risk of recurrence by restoring macrophage phagocytosis, antigen presentation, or immune memory.

It should be noted that immune responses are stage-dependent and vary among individuals. Excessive suppression of inflammatory responses may impair pathogen clearance, whereas excessive immune activation may aggravate tissue damage. Therefore, immunomodulatory materials should emphasize spatiotemporal control rather than simply pursuing a single direction of macrophage polarization.

### 4.4 Integrated osteogenic and angiogenic materials

After infection control, the repair of bone defects becomes a key determinant of functional recovery in patients. Materials such as hydroxyapatite, bioactive glass, collagen scaffolds, and biodegradable metals possess favorable osteoconductive or osteoinductive potential and can serve as carriers for antibacterial drugs or bioactive molecules (Ryan et al., 2019; Gupta et al., 2024; Bian et al., 2024). Metal ions such as copper, zinc, and magnesium not only exhibit certain antibacterial activity but also promote endothelial cell migration, osteogenic differentiation of bone marrow mesenchymal stem cells (BMSCs), or regulation of bone metabolism. Copper-eluting bioactive glass can simultaneously reduce infection and promote osteogenesis and angiogenesis (Ryan et al., 2019), whereas zinc-based biodegradable alloys and tannic acid-modified zinc materials achieve a favorable balance between antibacterial activity and osteogenesis (Jia et al., 2022; Zhao et al., 2023).

An ideal material for infected bone defect repair should combine mechanical support, biodegradability, osteogenic activity, and vascularization capacity. Three-dimensional printing technology enables the fabrication of personalized scaffolds according to defect morphology and allows regulation of cell adhesion, drug release, and new bone in growth through the control of pore size, porosity, and surface microstructure (Zhang et al., 2024; Bian et al., 2024; Li W. et al., 2025). However, in an infectious microenvironment, new bone formation often lags behind the demand for antibacterial treatment. Therefore, materials should avoid the premature release of osteogenic factors and should also prevent prolonged exposure to high doses of antibacterial components that may inhibit osteoblast activity.

## 5 Clinical translation challenges and perspectives

Although research on biomaterials for OM has advanced rapidly, clinical translation still faces multiple challenges. First, most studies remain at the level of *in vitro* experiments or small-animal models, which cannot fully recapitulate key factors in human chronic OM, such as sequestra, soft-tissue defects, diabetes, external fixation, or implant-associated conditions. Second, multifunctional materials often have complex structures involving multiple drugs, metal ions, nanoparticles, or externally triggered energy inputs. Their degradation products, long-term toxicity, and batch-to-batch consistency require systematic evaluation. In addition, although externally stimulated strategies such as photothermal therapy, sonodynamic therapy, microwave activation, and triboelectric approaches are innovative, their energy transmission in deep bone tissue, dose standardization, and clinical operational procedures remain to be further clarified.

Therefore, future research should first consider the construction of spatiotemporally responsive materials that preferentially release antibacterial components and eradicate biofilms at an early stage, followed by a transition toward anti-inflammatory, pro-angiogenic, and pro-osteogenic functions. In addition, material formats compatible with conventional surgical procedures, such as debridement, bone transport, internal fixation, or bone grafting, should be further developed. Examples include injectable hydrogels, moldable bone fillers, and personalized 3D-printed scaffolds. Meanwhile, animal models and evaluation systems that more closely resemble clinical scenarios are needed. In addition to conventional Micro-CT-based assessment of bone parameters, indicators such as mechanical strength, degree of vascularization, immune cell profiles, and recurrence rates should also be incorporated. Finally, for true clinical translation, greater attention should be paid to scalable manufacturing, cost control, and regulatory pathways, while avoiding excessive material complexity.

## 6 Conclusion

Emerging biomaterials provide the potential to transform OM treatment from local antibacterial therapy toward integrated regulation of infection control, immunomodulation, angiogenesis, and bone regeneration. Hydrogels, nanoparticles, and immune-engineered platforms can synergistically promote lesion healing through multiple mechanisms, including drug delivery, physical antibacterial strategies, gas therapy, ROS regulation, and macrophage reprogramming. In the future, OM biomaterials with genuine clinical value should not rely merely on the simple combination of multiple functions. Instead, they should be developed as intelligent therapeutic systems that are biodegradable, responsive to disease progression, and compatible with surgical treatment workflows.
